# Opinions of Patients, Families and Healthcare Professionals on Family Involvement in the Care of Patients Hospitalized in a Moroccan University Hospital: A Cross-Sectional Observational Survey

**DOI:** 10.3390/healthcare12181831

**Published:** 2024-09-13

**Authors:** Zohra Bahmane, Jihane Belayachi, Nawal Meknassi, Cortney Hughes Rinker, Redouane Abouqal, Naoufel Madani

**Affiliations:** 1 Acute Assessment Unit, Ibn Sina University Hospital, Mohammed V University, Rabat 10000, Morocco; jihanebelayachi@gmail.com (J.B.); nawalmeknassi19@gmail.com (N.M.); r.abouqal@um5r.ac.ma (R.A.); naoufelmad@gmail.com (N.M.); 2 Laboratory of Biostatistics, Clinical and Epidemiological Research, Faculty of Medicine and Pharmacy of Rabat, Mohammed V University, Rabat 10000, Morocco; 3 Global Affairs, George Mason University, Fairfax, VA 22030, USA; chughe13@gmu.edu

**Keywords:** Africa, attitudes, acute medicine, family involvement in care, opinions, patient and family centered care (PFCC), preferences

## Abstract

Opinion surveys on family participation in care in non-Western countries are rare. This study aims to assess the opinions of patients, families, and healthcare professionals regarding family involvement in care to identify their preferences and the associated factors. A cross-sectional survey was conducted over eight months involving 717 participants, using structured questionnaires at the Acute Assessment Unit of a university hospital in Morocco. Comparative analyses examined the association between participant characteristics and the preferences of care categories. Poisson regression was applied to determine factors associated with participant preferences. Attitudes toward family participation in care were positive, with an average score of 3.62 ± 0.43 on a 4-point Likert scale. Healthcare professionals were more favorable towards family participation, with an average of 10.6 ± 2.44 types of care, compared to 7.17 ± 1.96 for families and 5.71 ± 2.16 for patients. Participants’ opinions converged on a set of simple and less technical care tasks. Factors significantly associated with patient preferences in the final adjustment model (*p* < 0.05) included frailty, loss of autonomy, length of stay, and regular and continuous (day and night) family presence. This study highlights the strong support of health professionals, patients, and families for family participation in care. Understanding these preferences and related factors is essential to maximize family participation and develop a model of Patient And Family Centered Care adapted to the Moroccan context.

## 1. Introduction

Patient and Family Centered Care (PFCC) is an approach to care based on mutually beneficial partnerships between patients, family members, and healthcare professionals [1]. PFCC considers the family as a partner in the care of their hospitalized family members and as an integral part of the care team [2,3]. Family involvement in care is a key element of PFCC [4] and is recommended by international authorities due to its perceived benefits for patients, families, and caregivers [5,6]. Evidence from the literature indicates that healthcare professionals and family members agree on the need for partnership to provide optimal care and that encouraging family members to participate in care is essential because of the highly technical environment of an acute care unit [5].

In this context, experiments with liberalization of visiting hours, allowing increased family presence, are increasingly recognized as a means of improving PFCC [7]. Many studies support the establishment of open visitation policies in acute care units, with reported benefits for both patients and families [8].

Although encouraged by international organizations, family participation in care remains a vague concept and a non-standardized practice in adult acute care settings. Health professionals, patients, and families have nuances regarding the meaning of family participation in care and the forms that this participation could take [6,9]. In this regard, a scoping review identified five progressive components of family involvement in care along a continuum from relatively passive to active forms. These components are, respectively, presence, support, communication, decision-making, and contribution to care activities [10]. The literature has widely addressed the opinions of patients, families, and health professionals regarding the partially passive participation of the family in care, in particular, presence at the bedside, presence during invasive procedures, and participation in medical rounds. However, few opinion surveys have been able to assess the active participation of the family in care and the types of care in which families prefer to participate [5,10]. It is recognized that the views and attitudes of healthcare professionals towards the involvement of families in care are essential for the implementation of PFCC. The adoption of a positive attitude by health professionals towards the involvement of families promotes their active participation in the care process [6,11]. The exchange of information about patients’ medical histories and preferences helps to improve their medical care. In addition, information received by families on the progress of patients’ conditions encourages them to participate in care [12,13,14].

Results from a study in the United States found that nurses were more likely to invite families to participate in care. Nurses provided simple, non-invasive daily care rather than intimate (such as toileting) or technically skilled care (such as endotracheal tube suctioning) [15]. Results from another descriptive study in Saudi Arabia indicated that health professionals who opposed family presence during invasive procedures instead had positive attitudes toward family participation in routine care [9]. Other research suggests that most healthcare professionals and family members supported family involvement in essential care, with variations in preferred care activities [12,16].

In comparison with healthcare professionals and families, there appears to be a lack of literature describing family involvement in care from the perspective of adult patients hospitalized in acute care units [12]. However, the results of available studies revealed that patients were in favor of the active participation of their families in essential care [2,12,17].

Family involvement in care in adult care settings is not clearly delineated in the literature. Caregiving performed by families, as well as the preferred level of family involvement in direct care, has not been widely studied [5,6,18]. Several studies have attempted to identify acceptable care activities for patients, families, and providers in order to develop a care package as a model to facilitate the implementation of family involvement in care [17]. However, variations in preferences among different stakeholders make it difficult to identify a uniform list of care options for families [12]. Therefore, exploring the preferences of patients, families, and healthcare professionals to reach a consensus on appropriate care is an essential step to implementing family participation in care [6,16,19].

Most research on family involvement in adult patient care has been conducted in high-income countries and chronic care settings. In non-Western countries, despite the essential role that the family plays in the healthcare process, data addressing family participation in healthcare are rare [20,21,22,23,24].

In North African countries, the presence and support of family members for patients are based on traditions and cultures that sanctify family ties and social support between family members. Families feel responsible for their sick relatives and strive to fulfill this commitment by playing an important role in the care process [25,26,27,28].

In Morocco, a lower middle-income North African country, the participation of families in the care of their hospitalized family members is a usual practice, but is not formalized and remarkably less studied. Understanding the points of view of health professionals, patients, and families regarding family participation in care in an acute care context will help identify appropriate strategies to structure and sustain this practice into a model of Patient And Family Centered Care considered significantly effective in improving patient safety and the overall quality of care [12,15]. The objectives of this study, conducted in an acute assessment unit (AAU) of a Moroccan university hospital, were to evaluate the opinions of patients, families, and health professionals regarding participation in family care, identify their preferences regarding the type of care considered and determine the factors associated with these preferences.

## 2. Materials and Methods

### 2.1. Study Design

This cross-sectional observational study was conducted over eight months, from November 2018 to June 2019.

### 2.2. Study Setting

The study was conducted in the acute assessment unit (AAU) of a university hospital in Morocco.

The AAU is a continuous observation unit caring for adult patients primarily admitted from the emergency department and presenting with acute medical conditions. With a capacity of 30 beds, including 6 reserved for intensive care, the AAU ensures rapid and comprehensive assessment of patients and offers accelerated multidisciplinary care based on early warning scores. Family visits are officially scheduled from 4 p.m. to 7 p.m., but the AAU adopts an open visitation policy, allowing patients to be accompanied by their loved ones throughout their hospitalization. However, only one family member is allowed to stay with the patient outside of visiting hours.

### 2.3. Eligibility Criteria

Inclusion criteria were (1) all healthcare professionals working in the AAU, (2) patients admitted to the AAU with a hospital stay of at least 48 h, and (3) family members of these patients.

Exclusion criteria included refusal to participate, missing data, and hospital stays of less than 48 h. A hospital stay of more than 48 h was considered necessary to allow patients and their families to become familiar with the care offered and to facilitate the collection of opinions from them.

### 2.4. Sampling and Participants

#### 2.4.1. Healthcare Professionals

All eligible healthcare professionals (doctors, nurses, and support staff) working in the AAU were included through census sampling. It is important to note that support staff assist nurses with tasks like bathing, repositioning, and feeding and are not involved in cleaning activities, which are managed by outsourced services.

#### 2.4.2. Patients and Family Members

Patients admitted to the AAU who met the eligibility criteria, along with their most present family member (one per patient), were included through consecutive sampling.

### 2.5. Data Collection Methods and Tools

#### 2.5.1. Collection Tool

Data were collected from participants using a structured questionnaire. This questionnaire was prepared on the basis of existing literature and the researchers’ clinical experience. Content validity was assessed by critical care experts and statistical consultants. The data collection form consisted of two separate parts. The first part collected characteristics specific to each category (patients, families, and carers). The second part consisted of two opinion questionnaires (attitude scale and care scale), identical for all three categories (Supplementary S1). For the attitude scale, validity and reliability analyses showed good internal consistency, with Cronbach’s α’s of 0.86 for patients, 0.74 for families, and 0.76 for professionals. Exploratory factor analysis revealed a one-factor structure for each group of participants. For the care scale, exploratory factor analysis revealed a two-factor structure, with Cronbach’s α’s of 0.72 for patients, 0.78 for families, and 0.74 for professionals, attesting to adequate internal consistency for each group.

For healthcare professionals, data were collected using a self-administered questionnaire. For patients and their families, the data were collected by the interviewer, who completed the questionnaire face-to-face. The patients’ clinical data and outcome parameters were extracted from their medical records.

#### 2.5.2. Collection of Data

1. Characteristics of participants

Patients and their family members were interviewed separately. Only one family member per patient (the one most present) was interviewed.

- Patient characteristics

Sociodemographic characteristics included age, gender, marital status, number of living children, living arrangements, education level, geographical origin, and distance from the hospital.

Clinical characteristics included loss of autonomy assessed by the Activities of Daily Living (ADL) score [29], level of comorbidity assessed by the Comorbidities and Charlson Index (CCI) score [30], frailty prior to the acute episode leading to the hospitalization, assessed by the Clinical Frailty Scale (CFS) [31], the diagnosis upon admission and the probability of mortality assessed by the Simplified Acute Physiology Score II (SAPS II) calculated within the first 24 h of admission [32].

Outcomes parameters examined length of stay and mortality.

- Characteristics of family members

Included the presence of at least one family member accompanying the patient outside visiting hours, the number of family members who took it in turn to accompany each patient, the relationship of the family member most often present, the relationship of the second family member taking over, the regular presence of family members whether the family was present with the patient on a daily basis, and continuous presence when a family member was constantly present with the patient day and night.

- Characteristics of healthcare professionals

Data collected on healthcare professionals included age, gender, job title, and years of service.

2. Opinions of participants

- Participants’ attitudes toward family involvement in care

Participants’ attitudes toward family involvement in care were assessed using a Likert scale comprising nine items; each item allowed four possible responses. The score for each question ranged from 1 to 4. Family involvement in patient care: (1) is useful, (2) is essential, (3) provides psychological support, (4) can reduce family anguish and anxiety, (5) is not dangerous for patients, (6) does not interfere with the work of doctors and nurses, (7) does not make family hostile and aggressive towards doctors and nurses, (8) is good for patients, and (9) is good for families.

- Participants’ opinions regarding the types of care offered were collected using a list comprised of 14 types of care divided into three categories.
  ○ Dependency care comprises four types of care: Help with eating or changing clothes; support while walking or in a wheelchair; help changing positions or sitting on a chair; and accompaniment to the sink.
  ○ Intimate care groups together five types of care: Styling hair, shaving, or massage; accompaniment to the toilet; assistance while using the toilet; assistance in the shower; emptying urine bag or collecting urine to check for diuresis or for other testing.
  ○ Technical care comprises five care activities: Give medicines orally; perform mouth care; put on or take off nasal cannula for oxygen or the high oxygen concentration mask; take temperature; and perform capillary blood glucose test.

The participants identified the care they were in favor of according to the two response options proposed. The responses were “yes” (assigned value: 1) or “no” (assigned value: 0). The scores were calculated by adding the responses, giving a total score varying from 0 to 14. A higher score indicates a greater number of types of care that participants were in favor of.

The selection of the 14 types of care considered was based on the results of previous studies carried out in several healthcare contexts [12,16,23,33].

### 2.6. Statistical Analysis

Quantitative variables were expressed as median with quartiles or mean ± standard deviation, and qualitative variables as counts and percentages. Comparisons were made to assess the association between participant characteristics and categories of care. Symmetrically distributed continuous data were compared using the Student’s *t*-test for two independent samples and the ANOVA test for more than two independent samples. Continuous data with asymmetric distribution were compared using the Mann–Whitney U test for two independent samples and the Kruskal–Wallis test for more than two independent samples. The chi-square test or Fisher’s exact test were used for the comparison of qualitative variables. Univariate and multivariate Poisson regression was applied to determine the factors associated with the number of types of care that patients, families, and healthcare professionals were in favor of. Tests were considered significant when the level of significance (*p*-value) was less than 0.05. Statistical analysis was carried out using jamovi 2.5.5 software.

### 2.7. Ethical Considerations

The Biomedical Research Ethics Committee of Mohammed V University in Rabat gave its ethical approval for this study (reference number 41/15). All the necessary administrative authorizations were obtained from the responsible authorities at the university hospital. Participants were provided with information about the purpose and procedures of the study. Patients and their family members were informed that participation was voluntary and that refusal to participate would not affect their care. Free and informed consent was obtained from all participants prior to their involvement in the study. Participants were assured that anonymity and confidentiality of data were guaranteed. The study protocol is available in the appendices (Supplementary S2).

## 3. Results

**Figure 1:**
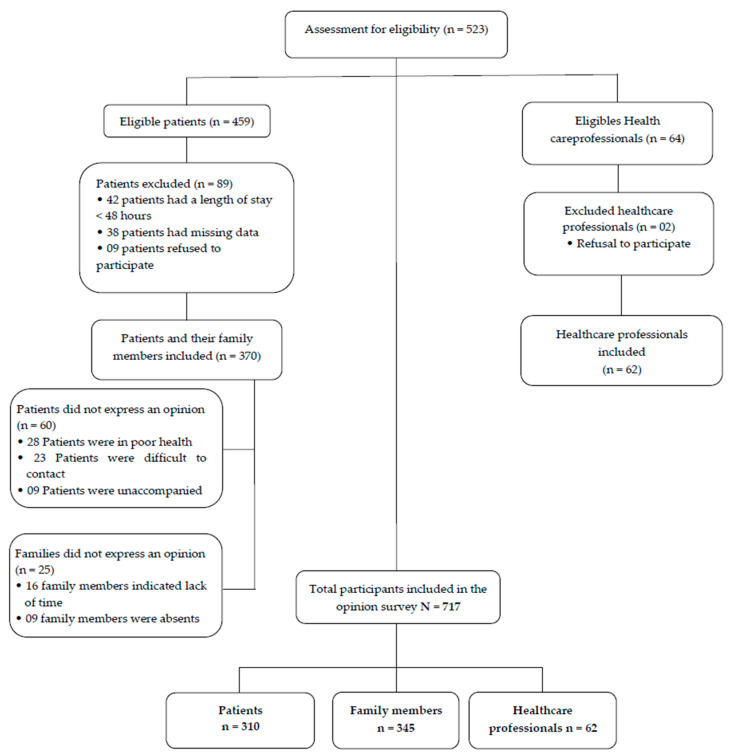
Number of participants included in the opinion survey (*n* = 717).

During the study period, out of a total of 64 health professionals working at the AAU, 62 were included in the study. Of the 459 patients admitted during the study period, 89 were excluded. A total of 370 patients and their family members were included (Figure 1). Of the 370 patients included and their family members most often present, 310 patients and 345 family members expressed their opinions regarding family participation in care and the types of care considered. In total, the investigator collected the opinions of 717 participants.

### 3.1. Characteristics of Patients, Family Members, and Healthcare Professionals

#### 3.1.1. Sociodemographic and Clinical Characteristics of Total Patients Included in the Study

The patients had a median age of 60 (45–68) years, and 213 (57.3%) of the patients were male. The majority of patients, 344 (97%), lived with family and 241 (65.1%) were married. The patients presented a total or partial loss of their autonomy with an ADL score on admission of 3.83 ± 2. Diagnoses on admission were dominated by cardiological emergencies (49.7%) and sepsis (20.8%). The median AAU length of stay and hospital length of stay were 8 [5,6,7,8,9,10,11,12,13] and 9 [6,7,8,9,10,11,12,13,14,15] days, respectively (Table 1).

#### 3.1.2. Characteristics of Family Members Accompanying Patients

Almost all patients, 361 (97.6%), were accompanied by their family members. A regular (daily) presence of the family was noted in 342 (94.7%) of the patients and a continuous presence (day and night) in 189 (52.4%) of the patients. The patients were mainly accompanied by a female family member (90.3%). The female members taking turns were almost double those of males (524 vs. 269). The family members most often present with their hospitalized family members were the spouse 132 (36.6%) and daughter 116 (32.1%) (Table 2).

Male patients were accompanied by their partners much more often than female patients (86.4% vs. 13.6%, *p* < 0.001). The daughter was the member most present among female patients (73.3% vs. 26.7%; *p* < 0.001), patients aged over 65 years (63.8% vs. 36.2%; *p* < 0.001), and patients with a partial or total loss of autonomy requiring the presence of a companion (82.8% vs. 17.2%; *p* < 0.001).

#### 3.1.3. Characteristics of Health Professionals

Table 3 shows the characteristics of health professionals. The average age of the health professionals was 33 ± 11.8 years, and 64.5% were female. More than half (54.8%) had seniority of more than one year.

### 3.2. Opinions of Patients, Families, and Health Professionals Regarding Family Participation in Care

All questionnaires distributed to healthcare professionals included in the study were completed and returned with a response rate of 100%. Of the 370 patients included in the study, 310 patients (84%) and 345 (96%) family members were interviewed and were able to express their opinions regarding family participation in care and the types of care considered for family participation in care.

#### 3.2.1. Participants’ Attitudes Toward Family Involvement in Care

Participants’ attitudes toward family involvement in care were assessed using a Likert scale. The overall mean score for participants was 3.62 ± 0.43 on the Likert scale (maximum 4), with mean scores of 3.80 ± 0.39 for patients, 3.91 ± 0.25 for families, and 3.14 ± 0.65 for healthcare professionals.

The majority of patients, families, and healthcare professionals strongly agreed with statements regarding the benefits of family participation, such as its usefulness, importance, and role in providing psychological support for patients and reducing anxiety for families. However, healthcare professionals were less likely to agree that family participation is not dangerous for patients (69.4%) and does not interfere with caregivers’ work (58%) compared to patients and families (Table 4).

Agreement among healthcare professionals that family participation does not hinder their work was associated with their experience and role. Younger professionals with less than one year of experience agreed more (71%) than those with more experience (52.9%, *p* = 0.028). Nurses were the least likely to agree (36.8%) compared to junior doctors (71%), senior doctors (60%), and support staff (60%, *p* = 0.026).

#### 3.2.2. Opinions of Participants Regarding Care Considered for Family Participation in Care

1. Participants’ preferences regarding the categories of care considered for family participation in care

**Figure 2:**
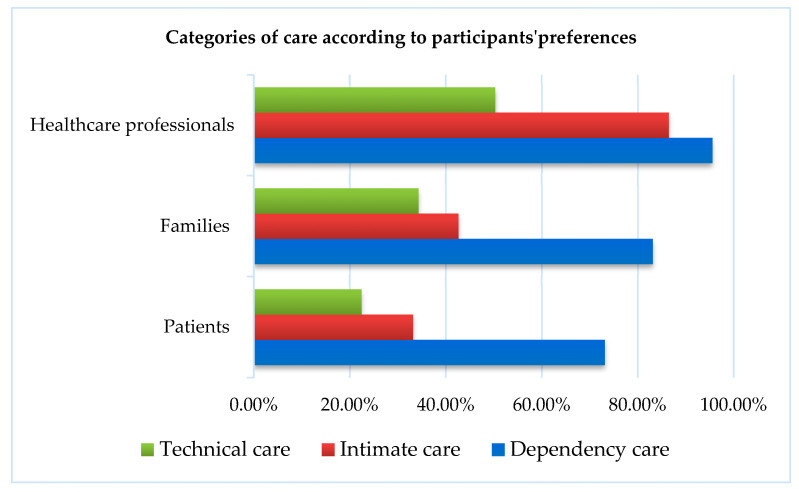
Categories of care according to participants’ preferences.

All three study groups—patients, families, and healthcare professionals—were in favor of family involvement in the three categories of care considered. Participants agreed on prioritizing dependency-related care, with an average fraction of care linked to this category of 73.1 ± 27.4% for patients, 82.8. ± 22.2% for families, and 95.6 ± 16% for health professionals. Intimate care came second in participants’ preferences, with an average fraction of care linked to this category of 33.2 ± 20.9% for patients, 42.4 ± 20% for families, and 86.5 ± 21.4% for health professionals. Technical care ranked last in the preferences of the three groups of participants, with an average fraction of care linked to this category of 22.5 ± 13.9% for patients, 34.4 ± 18.5% for families, and 50.3 ± 33.5% for health professionals (Figure 2).

Certain participant characteristics influenced their preferences. Female patients were more favorable towards family involvement in intimate care than male patients, with averages of 1.98 ± 1.06 vs. 1.45 ± 0.99 types of intimate care (*p* < 0.001). Patients over 65 preferred family involvement in technical care more than those under 65, with averages of 1.39 ± 0.63 vs. 1.05 ± 0.68 types of technical care (*p* < 0.001). Patients with a loss of autonomy (ADL score < 5) favored family involvement in all three categories of care more than those with retained autonomy: 3 [3, 4] vs. 2 [1–3] types of dependency care (*p* < 0.001), 1.91 ± 1.02 vs. 1.17 ± 0.93 types of intimate care (*p* < 0.001), and 1.27 ± 0.66 vs. 0.87 ± 0.66 types of technical care (*p* < 0.001).

Family members were equally supportive of dependency-related care for both older and younger patients (3.32 ± 0.87 vs. 3.34 ± 0.83, *p* = 0.863). However, they were more favorable towards providing intimate and technical care to patients over 65 than to those under 65, with averages of 2.31 ± 0.974 vs. 2.05 ± 1.02 types of intimate care (*p* = 0.027) and 1.85 ± 0.91 vs. 1.65 ± 0.92 types of technical care (*p* = 0.042).

2. Participants’ preferences regarding the types of care considered for family participation in care

Of the 14 types of care offered, the average number that participants were in favor of was 5.71 ± 2.16 for patients, 7.17 ± 1.96 for families, and 10.6 ± 2.44 for health professionals. Health professionals preferred to involve the family in the majority of the care offered. The health professionals were in favor of involving families in almost all dependency and intimate care offered. They were less favorable to the involvement of families in technical care. They preferred to involve families in simpler technical care such as mouth care (71%) and the administration of oral medication (69.4%).

Patients and families had similarities in the types of care they favored, with more types of care consistently favored by families than patients. For care related to dependency, the preferences of patients and families converged towards help with eating or dressing (96.5% vs. 99.7%) and support for walking or getting into a wheelchair (75.8% vs. 84.10%). For intimate care, patients and families opted for accompaniment to the toilet (72.30% vs. 74.20%). For technical care, the preferences of patients and families aligned with those of health professionals in favor of simple care. The administration of oral medication was the technical care most favored by patients (83.2%), families (93.6%), and health professionals (69.4%).

In summary, the participants’ preferences converged towards the following types of care: support for walking or getting into a wheelchair, change of position or getting into bed, support at the sink, giving food and drink or help with dressing, support to the toilet, massage or help with combing and shaving, and giving medication orally.

**Figure 3:**
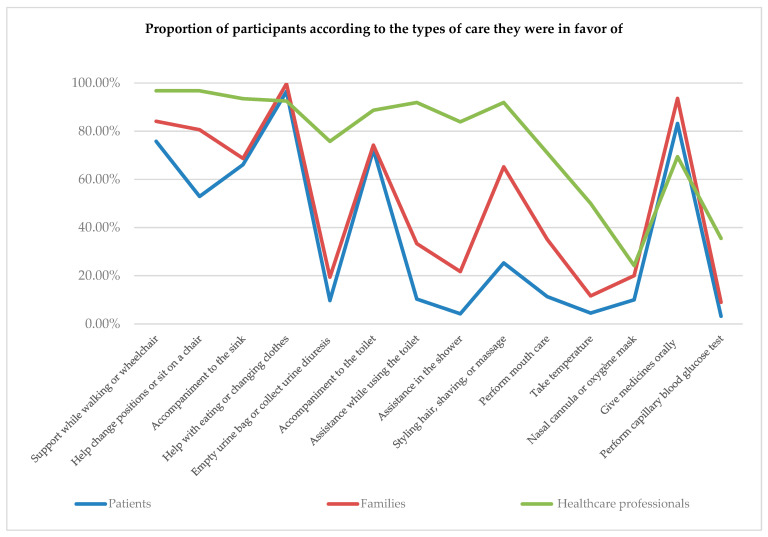
Preferences of patients, families, and healthcare professionals regarding the types of care considered.

Figure 3 shows the types of care preferred by participants. These preferences show that participants tend to favor simple care that provides direct physical assistance and support with basic daily activities and well-being.

### 3.3. Factors Associated with the Number of Types of Care That Patients, Families, and Health Professionals Were in Favor of Poisson Regression Identified Various Factors Associated with Participants’ Preferences for the Types of Care Considered

#### 3.3.1. Factors Associated with Patients’ Preferences

The univariate analysis revealed that certain patient and family member characteristics were significantly associated with the number of types of care that patients were in favor of.

In the multivariate analysis, the number of types of care patients favored remained associated with clinical variables and outcome parameters, as well as the nature of family presence. Specifically, patients with pre-existing frailty (high clinical frailty score) (aRR = 1.05; 95% CI: 1.00–1.10), those with a loss of independence on admission (ADL score < 6) (aRR = 0.94; 95% CI: 0.91–1.96), and those with longer stays in the AAU (aRR = 1.00; 95% CI: 1.00–1.01) favored more types of care. Additionally, as shown in Table 5, a regular family presence increased the number of types of care patients favored by 64%, while continuous family presence (day and night) increased this by 21%.

#### 3.3.2. Factors Associated with Families’ Preferences

The univariate analysis showed that the number of types of care to which families were favorable was significantly associated with the sociodemographic and clinical characteristics of the patients, particularly the female sex of the patients (RR = 1.10; 95% CI: 1.02–1.19) and loss of autonomy assessed by the ADL score at admission (RR = 0.97; 95% CI:0.95–0.99). The number of types of care that families were in favor of was also associated with the characteristics of family members, notably the number of female companions taking turns per patient (RR = 1.07; 95% CI: 1.02–1.13) (Table 6). However, the multivariate analysis indicated that no variable was significantly associated with the number of types of care preferred by families.

#### 3.3.3. Factors Associated with Healthcare Professionals’ Preferences

The Poisson regression analysis presented in Table 7 showed no significant association between the characteristics of healthcare professionals and the number of types of care they favored in both univariate and multivariate analyses.

## 4. Discussion

The objective of our study was threefold: to evaluate the opinions of patients, families, and health professionals regarding family participation in care; to identify the preferences of the three categories of participants regarding the types of care considered; and to determine the factors influencing the preferences of each category of participants. This study is the first research of its kind to explore the opinions of patients, families, and health professionals regarding family participation in care in a North African country.

This study, carried out in an open-door acute assessment unit of a Moroccan university hospital, demonstrated that healthcare professionals, patients, and their family members were supportive of family involvement in care. Similar to the results of previous studies, the three study groups were unanimous on the importance of family participation in care and its beneficial effects [2,5,12,16,17]. Patients and families viewed family involvement in care as a natural role for the family and safe for the patient. This attitude can be explained by the simplicity of the care offered and the explanations provided to each member of the family before becoming involved in care [12,34]. In addition, volunteering to participate in care relieves families from providing care that they consider dangerous or difficult to carry out [12,35]. Authorized to stay with their hospitalized family members, families did not feel like intruders. They were convinced that their presence and participation in the care were contributory to the medical care of their hospitalized family members. These results contrast with previous studies which demonstrated that many family members report that they felt “inconvenient” or feared they hindered providers, which affected their participation [5].

As in many low-resource countries, the nurse–patient ratio at the AAU is high (1:10), and the involvement of families in care becomes a means of compensating for the shortage of health professionals [36,37,38,39]. Compared with the restricted visitation policy adopted by the emergency department, where the majority of patients admitted to the AAU come from, the open visitation policy adopted at the AAU was highly appreciated by patients and their families. Patients and families considered open family visits to be an essential condition for the success of family involvement in care [12,38,40].

Most patients were accompanied by family members who supported their participation in the proposed care. The family members most present were the spouse and the daughter [25,28,41]. The most vulnerable patients were more supportive of involving their families in care. These patients were mainly accompanied by their daughters. These results correspond to previous studies, which mentioned that family ties could influence willingness to participate in care [42,43,44].

The category of care most favorably viewed by all three study groups was dependency care. This result seems consistent with the majority of Western and non-Western studies [38,39,40,41]. The category of intimate care ranks second among participants’ preferences, reflecting the cultural specifics of non-Western countries, which place higher value on family involvement in these types of care. In contrast, in Western countries, participation in intimate care is often perceived as an intrusion and is generally avoided [24,36,38]. Furthermore, in Arab–Muslim countries like Morocco, cultural and religious values strongly emphasize modesty. Consequently, patients, families, and healthcare professionals prefer that families be involved in intimate care, in line with these cultural norms.

While families viewed their participation in dependency and intimate care as a natural role, they perceived technical care as an area specific to healthcare professionals and preferred that they be invited to participate in this type of care [12,34,35]. However, health professionals were reluctant to involve families in technical care. As supported by the literature, family involvement in technical care raises concerns about patient safety and healthcare professional liability [9,15,17,39,43]. Several studies have highlighted the need for education, training, and guidance of health professionals to ensure family participation in care is conducted safely [9,12,34]. In addition, health professionals preferred to involve families in dependency and intimate care to have more time to concentrate on the technical care that they appropriate and consider as their main activity. Similar to other studies, the care activities preferred by patients, families, and health professionals for family participation were daily activities and simple, less technical care, including assistance with feeding and dressing, support with walking, assistance with the toilet, and administration of oral medications [15,16,24,44,45].

This study provided a portrait of the factors influencing participants’ preferences regarding the types of care considered. Female patients showed a greater preference for involving their families in more care. A plausible explanation for this is that many patients are accompanied by female family members, and they often prefer to receive care from people of the same sex. In turn, female family members were more willing to participate in more suggested caregiving. This may be explained by the role traditionally assigned to women in the care and support of the family, particularly the daughters who accompany their relatives.

It is well established that family characteristics influence the willingness of family members to actively participate in care [16]. Patients with frailty before the acute episode were in favor of involving their families in a high amount of care, probably due to their pre-existing frailty and the usual help received from their relatives for certain daily activities. Similarly, patients with a loss of independence at admission were highly supportive of this participation due to their increased need for assistance with basic activities and overall well-being.

Unlike some intensive care unit studies that limit family participation in care due to patient condition severity [16,41], this study suggests that in the AAU, severity may, on the contrary, encourage greater family involvement in care. This may be attributed to the fact that patients in the AAU are not on mechanical ventilation, making family involvement in care more feasible and potentially beneficial. Patients with longer AAU stays favored increased family involvement, which is consistent with findings indicating that longer stay is a factor favoring family involvement in care [41]. Additionally, patients with regular or continuous family presence were more supportive of increased involvement of their families in care. This preference may stem from the availability and responsiveness of family members to patients’ needs, as well as the high severity of these patients’ health conditions, requiring constant support. Indeed, patients with a continuous family presence presented higher frailty, lower autonomy, and higher severity scores [33].

This study also showed that health professionals were reluctant to involve the family in technical care. As already mentioned, this reluctance could be due to the legal responsibility of healthcare providers towards patients and the desire to avoid the risks involved when the family participates in care [10,15,35,46]. In addition, patient acuity, staff shortages, and the absence of written procedures relating to family participation in care are reported to be factors limiting the invitation of families to care [9,15,35].

These results highlight the importance of taking into account, when planning care and involving the family in the AAU, (1) the individual characteristics of patients, such as their autonomy and frailty, which influence their care needs and their receptivity to family involvement, (2) family dynamics including the availability, preferences, and abilities of family members to participate in the patient’s care, and (3) institutional factors such as written procedures regarding participation from family to care, staff coordination, training, and educational approaches to involve the family in care. The consideration of all these elements aims to involve the family in care while ensuring patient safety and the quality of care.

### 4.1. Strengths and Limitations

A major strength of this study is that it is the first to investigate opinions on family involvement in care in a North African country. Notably, the study stands out by evaluating the views of all key stakeholders—healthcare professionals, patients, and families—unlike most previous studies, which often focus solely on the perspectives of families and/or healthcare professionals. Additionally, this research is among the few that examine opinions on active family involvement in an acute care setting.

Furthermore, the substantial sample size of 717 participants adds significant robustness to the findings and enhances the representativeness of the study population. The use of structured questionnaires during interviews is another notable strength, ensuring consistent data collection, minimizing interviewer bias, and enabling a more reliable comparative analysis of responses. As a result, this study makes a valuable contribution to the existing literature on family involvement in care and offers a solid foundation for future research across a broader range of contexts.

However, the study also has several limitations. It is a single-center study conducted in an AAU of a large Moroccan university hospital, which may limit the generalizability of the findings to other Moroccan or similar acute care settings. Another limitation is that while the structured questionnaires provided consistency, the study design relied on interviews, which could introduce social desirability bias, where participants might provide answers they believe are expected rather than their true opinions. Additionally, the cross-sectional design of the study might not capture changes in opinions over time. Another limitation is the lack of assessment of satisfaction with family involvement in care, which is crucial for understanding the effectiveness and impact of such involvement. This aspect will be addressed in future research.

### 4.2. Prospects

This study serves as a preliminary step towards identifying a care model suitable for family participation, with particular attention to technical care, which should be simple and selected by consensus among the various stakeholders. To structure family involvement and expand the range of care in which they can participate, it is essential to implement appropriate training and supervision for families. Additionally, educational strategies must be developed to guide families in their involvement in specific care activities. In the future, it would be pertinent to test the integration of family participation within a care model such as Patient and Family Centered Care. Further research could also explore the long-term impacts of this participation on care outcomes and patient well-being.

## 5. Conclusions

In this AAU, healthcare professionals, patients, and their families were in favor of involving families in care, with a particular focus on dependency-related care. The open visiting policy implemented by the AAU encourages the presence of families and supports their involvement in care. Participants’ opinions converge on a set of simple, less technical care activities. Family involvement in care was significantly associated with individual patient characteristics, such as frailty and autonomy, with family dynamics, such as regular or continuous family presence, and with institutional factors, such as length of stay.

Given these encouraging results, the AAU is well placed to serve as a pilot site for the implementation of a Patient And Family Centered Care model. This initiative will ensure the structured and sustained integration of families into care, particularly that which corresponds to the preferences identified in this study. Implementation of this model in the AAU could provide valuable information and data for wider application in similar acute care settings, ideally multicenter, making the AAU a benchmark for innovative care practices.

## Figures and Tables

**Table 1:** Characteristics of patients included in the study (*n* = 370).

Characteristics	*n* (%)
Sociodemographic variables	
Age, median [IQR *], years	60 [45–68]
Male gender	212 (57.3)
Female gender	158 (42.7)
Marital status	
Married	241 (65.1)
Not married	129 (34.9)
Number of children, median [IQR *]	3 [1–5]
Living situation	
Lives with family	344 (93)
Does not live with family	26 (7)
Distance between hospital and place of residence	
Within the city of Rabat (0 à 20 km)	207 (55.9)
More than 20 km	163 (44.1)
Level of education	
None or primary	287 (77.6)
Secondary	58 (15.7)
University	25 (6.8)
Clinical variables and outcome parameters	
Frailty Scale, mean ± standard deviation	3.2 ± 1.1
ADL * score at admission, mean ± standard deviation	3.83 ± 2
Charlson score, median [IQR *]	2 [1–4]
SAPS II *, mean ± standard deviation	21.5 ± 9.4
Diagnosis	
Cardiac emergencies	184 (49.7)
Sepsis	74 (20)
Other	112 (30.3)
LOS median [IQR*]	
In acute assessment unit	8 [5–13]
In hospital	9 [6–15]

* IQR: Interquartile range. ADL: Activities of Daily Living. SAPS II: Simplified Acute Physiology Score.

**Table 2:** Characteristics of family members accompanying their hospitalized family members.

Characteristics	*n* (%)
Age, median [IQR *], years	45 [35–53]
Total number of family members	793 (100)
Female	524 (66.1)
Male	269 (33.9)
Number of family members who take turns per patient, mean ± SD *	2 ± 0.8
Only one family member present in the 361 accompanied patients More than one family member present	65 (18)
More than one family member present in the 361 accompanied patients	296 (82)
Relationship of family member most often present	361 (100)
Spouse	132 (36.6)
Daughter	116 (32.1)
Other family member	83 (23)
Son	24 (6.6)
Unrelated	6 (1.6)
Relationship of second family member who takes over	296 (82)
Other family member	109 (36.8)
Daughter	82 (27.7)
Son	78 (26.4)
Spouse	15 (5.1)
Unrelated	12 (4.1)
Daily presence of a family member in the 361 accompanied patients	342 (94.7)
Continuous presence of a family member in the 361 accompanied patients	189 (52.4)

* IQR: Interquartile range. SD: standard deviation.

**Table 3:** Characteristics of healthcare professionals (*n* = 62).

Characteristics	*n*%
Age, median [IQR *], years	26.5 [24–42]
Sexe	
Male	22 (35.5)
Female	40 (64.5)
Profession	
Senior doctor	5 (8.1)
Junior doctor	28 (45.2)
Nurse	19 (30.6)
Support staff	10 (16.1)
Years in practice	
Less than one year of practice	28 (45.2)
More than one year of practice	34 (54.8)

* IQR: Interquartile range.

**Table 4:** Comparison of patients, families, and healthcare professionals’ attitudes toward family involvement in care (*n* = 717).

Attitudes	Patients (*n* = 310)		Families (*n* = 345)		Healthcare Professionals (*n* = 62)	
	Agree *n* (%)	Disagree *n* (%)	Agree *n* (%)	Disagree *n* (%)	Agree *n* (%)	Disagree *n* (%)
Family involvement in patient care is useful	310 (100.0)	-	345 (100.0)	-	60 (96.8)	2 (3.2)
Family involvement in patient care is essential	300 (96.8)	10 (3.2)	344 (99.7)	1 (0.3)	57 (91.9)	5 (8.1)
Family involvement in patient care provides psychological support	308 (99.4)	2 (0.6)	345 (100.0)	-	60 (96.8)	2 (3.2)
Involvement in care can reduce family anguish and anxiety	304 (98.1)	6 (1.9)	345 (100.0)	-	54 (87.1)	8 (12.9)
Family participation in care is not dangerous for patients	307 (99)	3 (1)	341 (98.8)	4 (1.2)	43 (69.4)	19 (30.6)
Family involvement in care does not interfere with the work of doctors and nurses	308 (99.4)	2 (0.6)	343 (99.4)	2 (0.6)	36 (58)	26 (42)
Family involvement in care does not make them hostile and aggressive towards doctors and nurses	307 (99)	3 (1)	339 (98.2)	2 (0.6)	53 (85.5)	9 (14.5)
Family involvement in care is good for patients	309 (99.7)	1 (0.3)	345 (100.0)	-	58 (93.5)	4 (6.5)
Family involvement in care is good for families	296 (95.5)	14 (4.5)	342 (99.1)	3 (0.9)	52 (83.9)	10 (16.1)

**Table 5:** Poisson regression analysis of the association between patient characteristics, family characteristics, and the number of care patients were in favor of Significant *p*-values (less than 0.05) are highlighted in bold.

Variables	Univariate			Multivariate		
	RR *	95% CI *	*p* Value	RR Adjusted	95% CI	*p* Value
Patient characteristics						
Sociodemographic variables						
Sex						
Female	1.19	[1.09–1.30]	**<0.001**	1.01	[0.89–1.14]	0.841
Male	Réf					
Age						
Age > 65	1.19	[1.09–1.31]	**<0.001**	1.07	[0.94–1.22]	0.291
Age < 65	Réf					
Number of living children	1.02	[1.01–1.04]	**0.001**	1	[0.97–1.02]	0.976
Marital status						
Married	1.12	[0.99–1.27]	0.065	1.2	[0.99–1.45]	0.062
widowed	1.39	[1.18–1.65]	**<0.001**	1.16	[0.92–1.47]	0.268
Divorced	1.2	[0.91–1.57]	0.183	1.27	[0.95–1.70]	0.199
single	Réf					
Education						
Primary	0.93	[0.84–1.03]	0.165	1.03	[0.92–1.17]	0.525
secondary	0.89	[0.79–1.01]	0.085	1.1	[0.95–1.27]	0.195
university	0.96	[0.81–1.14]	0.654	1.12	[0.93–1.35]	0.229
No education	Réf					
Distance	1	[0.99–1.00]	0.981	1	[0.99–1.00]	0.994
Clinical variables and outcome parameters						
Frailty scale score before acute episode	1.12	[1.07–1.17]	**<0.001**	1.05	[1.00–1.10]	**0.043**
ADL score on admission	0.9	[0.89–0.92]	**<0.001**	0.94	[0.91–1.96]	**<0.001**
Charlson score	1.04	[1.02–1.07]	**<0.001**	1	[0.97–1.05]	0.653
SAPS II * SCORE	1.01	[1.01–1.02]	**<0.001**	1	[0.99–1.00]	0.884
LOS * in acute assessment unit	1.01	[1.01–1.02]	**<0.001**	1	[1.00–1.01]	**0.004**
Chronic disease						
Presence of chronic disease	1.13	[1.04–1.24]	**0.004**	1.02	[1.91–1.14]	0.721
No chronic disease	Réf					
Diagnosis						
sepsis	1.17	[1.05–1.30]	**0.005**	1.05	[0.93–1.18]	0.381
Other diagnosis	Réf					
Characteristics of families						
Number of male companions	0.92	[0.86–0.98]	**0.010**	0.97	[0.90–1.04]	0.495
Number of female companions	1.07	[1.01–1.13]	**0.016**	0.98	[0.91–1.05]	0.579
Relationship						
Son	1.12	[0.75–1.66]	0.585	0.91	[0.59–1.41]	0.688
Daughter	1.32	[0.91–1.90]	0.142	0.95	[0.63–1.43]	0.826
Spouse	1.05	[0.73–1.52]	0.776	0.92	[0.62–1.39]	0.721
Other member	1.12	[0.77–1.62]	0.549	1.09	[0.74–1.61]	0.644
No family relationship	Réf					
Daily presence of family						
Regular presence	2.05	[1.57–2.68]	**<0.001**	1.64	[1.21–2.23]	**0.001**
Irregular presence	Réf					
Family present (day and night)						
Continuous presence	1.46	[1.34–1.60]	**<0.001**	1.21	[1.09–1.35]	**<0.001**
Discontinuous presence	Réf					

* ADL: Activities of Daily Living.95% CI: 95% Confidence Interval. LOS: Length of Stay. RR: Risk Ratio. SAPS II: Simplified Acute Physiology Score.

**Table 6:** Poisson regression analysis of the association between patient characteristics, family characteristics, and the amount of care to which families were in favor Significant *p*-values (less than 0.05) are highlighted in bold.

Variables	Univariate			Multivariate		
	RR *	95% CI *	*p* Value	RR Adjusted	95% CI	*p* Value
Patient characteristics						
Sociodemographic variables						
Sex						
Female	1.1	[1.02–1.19]	**0.015**	1.05	[0.94–1.18]	0.330
Male	Réf					
Age						
Age > 65	1.07	[0.98–1.16]	0.112	1.04	[0.92–1.18]	0.490
Age < 65	Réf					
Number of living children	1	[0.99–1.02]	0.581	0.99	[0.79–1.02]	0.766
Marital status						
Married	1.12	[1.01–1.25]	**0.021**	1.07	[0.92–1.24]	0.335
widowed	0.94	[0.83–1.06]	0.347	0.91	[0.76–1.11]	0.387
Divorced	1.04	[0.83–1.32]	0.694	1.01	[0.78–1.31]	0.891
Single	Réf					
Education						
Primary	1.02	[0.93–1.13]	0.577	1.08	[0.97–1.21]	0.149
Secondary	0.92	[0.82–1.05]	0.225	1.01	[0.87–1.18]	0.822
University	0.96	[0.82–1.14]	0.654	1.02	[0.85–1.23]	0.762
No education	Réf					
Distance	1	[0.99–1.00]	0.871	1	[0.99–1.00]	0.883
Clinical variables and outcome parameters						
FS score before acute episode	1.02	[0.98–1.06]	0.206	0.99	[0.94–1.04]	0.776
ADL score on admission	0.97	[0.95–0.99]	**0.005**	0.98	[0.95–1.00]	0.116
Charlson score	1.02	[1.00–1.04]	**0.034**	1	[0.97–1.04]	0.726
SAPS II * SCORE	1.01	[1.00–1.01]	0.075	0.99	[0.99–1.01]	0.134
LOS * in acute assessment unit	1	[1.00–1.01]	**0.029**	1	[0.99–1.01]	0.134
Chronic disease						
Presence of chronic disease	1.07	[0.98–1.16]	0.098	1.01	[0.91–1.13]	0.763
No chronic disease	Réf					
Diagnosis						
Sepsis	1.06	[0.96–1.16]	0.260	0.99	[0.89–1.10]	0.895
Other diagnosis	Réf					
Characteristics of families						
Number of male companions	0.97	[0.91–1.03]	0.321	1.01	[0.94–1.08]	0.720
Number of female companions	1.07	[1.02–1.13]	**0.010**	1.05	[0.99–1.13]	0.090
Relationship						
Son	0.81	[0.55–1.19]	0.288	0.79	[0.53–1.28]	0.292
Daughter	0.93	[0.65–1.33]	0.713	0.79	[0.53–1.18]	0.261
Spouse	0.87	[0.61–1.24]	0.439	0.82	[0.55–1.21]	0.322
Other member	0.9	[0.63–1.29]	0.583	0.88	[0.60–1.30]	0.544
No family relationship	Réf					
Daily presence of family						
Regular presence	1.24	[0.97–1.57]	0.077	1.13	[0.86–1.49]	0.351
Irregular presence	Réf					
Family present (day and night)						
Continuous presence	1.08	[0.99–1.17]	0.070	0.99	[0.90–1.11]	0.992
Discontinuous presence	Réf					

* ADL: Activities of Daily Living. 95% CI: 95% Confidence Interval. FS: Frailty Scale. LOS: Length of Stay. RR: Risk Ratio. SAPS II: Simplified Acute Physiology Score.

**Table 7:** Poisson regression analysis of the association between healthcare professionals’ characteristics and the number of care that healthcare professionals were in favor of.

Variables	Univariate			Multivariate		
	RR *	95% CI *	*p* Value	RR Adjusted	95% CI	*p* Value
Age	0.99	[0.99–1.00]	0.277	0.92	[0.98–1.00]	0.129
Sex						
Male	1.03	[0.87–1.20]	0.757	1.11	[0.92–1.34]	0.264
Female	Réf					
Years of exercise						
>1 year	0.95	[0.82–1.12]	0.588	1.15	[0.89–1.48]	0.267
<Less than 1 year	Réf					
Job title						
Senior doctor	1.02	[0.77–1.36]	0.865	1.08	[0.74–1.56]	0.683
Nurse	0.84	[0.70–1.02]	0.072	0.81	[0.64–1.04]	0.094
Support staff	0.91	[0.72–1.14]	0.404	0.92	[0.66–1.28]	0.623
Junior doctor	Réf					

* 95% CI: 95% Confidence Interval. RR: Risk Ratio.

## Data Availability

The data for this study are available and can be made available if necessary.

## Supplementary Materials

The following supporting information can be downloaded at https://www.mdpi.com/article/10.3390/healthcare12181831/s1: Supplementary Materials S1: Questionnaire for Patients, families and healthcare professionals regarding Family Involvement In Care Of Patients Hospitalized At the Acute Assessment Unit (AAU); Supplementary Materials S2: Study protocol.
