# Supplementary material for: Opinions of Patients, Families and Healthcare Professionals on Family Involvement in the Care of Patients Hospitalized in a Moroccan University Hospital: A Cross-Sectional Observational Survey
Source: Healthcare (Basel). 2024 Sep 13;12(18):1831. doi: 10.3390/healthcare12181831 (PMC11431572; doi:10.3390/healthcare12181831)
Supplement: Supplementary file 1 [file healthcare-12-01831-s001.zip › healthcare-3136721-Supplementary update.pdf]

Questionnaire for Patients Regarding Family Involvement In Care Of Patients Hospitalized At the Acute Assessment Unit (AAU)

Patient code: ………………

Family member code: ………..

We would like to know your opinion on family participation in the care of hospitalized patients. This questionnaire is anonymous and will take you a few minutes to answer. Your responses will remain strictly confidential.

1. SOCIODEMOGRAPHIC CHARACTERISTICS:

- **Age**……………..years < 65 year ☐ > 65 year ☐
- **Sex**: M ☐ F ☐
- **Origin**: Urban ☐ Rural ☐
- **City**……………………..….… Number of Km / Rabat….. …..
- **Marital Status**: Married ☐ Widowed ☐ Divorced ☐ single ☐
- **Number of living children**…………..
- **Living conditions**: Lives alone ☐ With spouse only ☐ With family ☐
  Retirement home ☐ Homeless ☐ Social isolation ☐
- **Educational level**: No Formal Education ☐ Primary ☐ Secondary ☐ Higher Education ☐

2. CLINICAL FEATURES :

Admission Source :

- **Provenance**: Emergencies Department ☐ Other service ☐ specify ……………
- **Deadline :** Emergencies Department – AAU < 24 H ☐ > 24 H ☐

Medical History:

- **Previous hospitalizations** ☐ where ?.........
- **Chronic illness(es)**: ☐ Which:
  Heart failure ☐ Hypertension ☐ Diabetes ☐
  Chronic Respiratory Failure ☐ Chronic Renal Failure ☐ Cirrhosis ☐
  Cancer ☐ Others :…………
- **Chronic treatment in progress** ☐

Questionnaire for Patients Regarding Family Involvement In Care Of Patients Hospitalized At the Acute Assessment Unit (AAU)

Clinical Parameters at Admission:

- **FS before the acute episode: ………..**
- **ADL before acute episode: ………**
- **ADL on admission: ..……………**
- **Reduced food intake for…………days before admission** □
- **Charlson: …..……..**
- **SAPSII……………**
- **Diagnosis……………..**

Outcome Parameters

- **Exit**: Direct □ Against Medical Advice □ At The Request Of The Family □ Transfer □ where ?..............
- **Length Of Stay (LOS) to**:
  - AAU: ……..days LOS ≤ 24 hours□
  - Hospital: ……..days
  - Emergencies Department : ……………days
- **Decease** :
  - AAU □
  - Hospital □

3. FAMILY PRESENCE

- Need for the presence of a family member (ADL<5): Yes □ No □
- Presence of at least one family member: Yes □ No □

4. PATIENT OPINION REGARDING FAMILY INVOLVEMENT IN CARE

- Opinion expressed □ Opinion not expressed □
- Reason why the opinion was not expressed: Short LOS □ Refusal □ Dementia □ Psychosis □ Disorders of consciousness □ Very weathered □ Difficult communication □ Deafness □ Mutism □ Other □ To specify ……………………………

Questionnaire for Patients Regarding Family Involvement In Care Of Patients Hospitalized At the Acute Assessment Unit (AAU)

I/ Patients ‘attitudes toward family involvement in care:

What do you think about family involvement in the care of hospitalized patients?

1. Involvement of families in patient care is useful. You are :

   Completely disagree □ Disagree □ Agree □ Completely agree□

2. Family participation in patient care is essential. You are :

   Completely disagree □ Disagree □ Agree □ Completely agree□

3. Does your family's participation in care provide psychological support for you?

   Completely disagree □ Disagree □ Agree □ Completely agree□

4. Involving your family in care can reduce their anxiety:

   Completely disagree □ Disagree □ Agree □ Completely agree □

5. Your family's participation in care is not dangerous for you:

   Completely disagree □ Disagree □ Agree □ Completely agree □

6. Your family's participation in care does not interfere with the work of doctors and nurses:

   Completely disagree □ Disagree □ Agree □ Completely agree □

7. In general, your family's involvement in care does not make them hostile and aggressive toward doctors and nurses:

   Completely disagree □ Disagree □ Agree □ Completely agree □

8. You consider that your family's participation in care is a good thing for you:

   Completely disagree □ Disagree □ Agree □ Completely agree □

9. You consider that participation in care is a good thing for your family:

   Completely disagree □ Disagree □ Agree □ Completely agree □

Questionnaire for Patients Regarding Family Involvement In Care Of Patients Hospitalized At the Acute Assessment Unit (AAU)

II/ Patients' opinions regarding the types of care considered

Could you specify the care that you are in favor of being carried out by your family?

Category of care	Type of care	Yes	No	score
**Dependency Care**	Help with eating or changing clothes			
	Help with walking or using a wheelchair			
	Help with position changes or sitting in a chair			
	Accompaniment to the sink			
**Intimate care**	Hair dressing, shaving or massage,			
	accompaniment to the toilet			
	help with using the toilet			
	help in the shower			
	emptying the urine bag or collecting urine for diuresis or other tests			
**Technical Care**	Administer medications orally,			
	Perform mouth care			
	put on or take off the nasal cannulas for oxygen or the high concentration oxygen mask			
	take the temperature			
	perform a hair blood glucose test			
Total score:				

Questionnaire for Family Members Regarding Family Involvement In Care Of Patients Hospitalized At the Acute Assessment Unit (AAU)

Patient code: ………………

Family Member Code: ………………

We would like to know your opinion on family participation in the care of hospitalized patients. This questionnaire is anonymous and will take you a few minutes to answer. This information are strictly confidential.

1. INFORMATION ABOUT THE MOST PRESENT FAMILY MEMBER

- **Relationship**: ………………………………………
- **Age :**……….. years
- **Sex**: M □ F □

2. INFORMATION ABOUT FAMILY MEMBERS TAKING TURNS

- **Number of family members taking turns**:…………..
  - **Number of male family members:**……..
  - **Number of female family members:**…….
- **Relationship of the second member of the family who often takes over**:…………………...........................……………………………

3. PRESENCE OF FAMILY MEMBERS:

- **Family presence during the day : YES** □ **No** □
  - **Regular presence (Every day) :** □ Morning After noon □
  - **Irregular (not-daily) presence:** □
- **Family presence day and night :** Yes □ No □
  - **Continued presence**: □ Morning □ After noon □ night □
  - **Discontinued presence:** □ Morning □ After noon □ night □

OPINION OF THE MOST PRESENT FAMILY MEMBER REGARDING FAMILY PARTICIPATION IN CARE

- Opinion expressed □
- Opinion not expressed □ Why: Short LOS □ Denied □ Not available □ Social isolation □ Other □ To specify ……………………………

Questionnaire for Family Members Regarding Family Involvement In Care Of Patients Hospitalized At the Acute Assessment Unit (AAU)

I/ Family members 'attitudes toward family involvement in care:

What do you think about family involvement in the care of hospitalized patients?

1. Involvement of families in patient care is useful You are :

   Completely disagree □ Disagree □ Agree □ Completely agree□

2. The participation of families in patient care is essential. You are :

   Completely disagree □ Disagree □ Agree □ Completely agree□

3. Does your participation in care provide psychological support to your hospitalized member?

   Completely disagree □ Disagree □ Agree □ Completely agree□

4. Your participation in care can reduce your anxiety:

   Completely disagree □ Disagree □ Agree □ Completely agree□

5. Your participation in care is not dangerous for your hospitalized family member:

   Completely disagree □ Disagree □ Agree □ Completely agree□

6. Your participation in care does not interfere with the work of doctors and nurses:

   Completely disagree □ Disagree □ Agree □ Completely agree□

7. In general, your participation in care does not make you hostile or aggressive towards doctors and nurses:

   Completely disagree □ Disagree □ Agree □ Completely agree□

8. You consider that your participation in care is a good thing for your hospitalized family member:

   Completely disagree □ Disagree □ Agree □ Completely agree□

9. You consider that participation in care is a good thing for you:

   Completely disagree □ Disagree □ Agree □ Completely agree□

Questionnaire for Family Members Regarding Family Involvement In Care Of Patients Hospitalized At the Acute Assessment Unit (AAU)

II/ Family members' opinions regarding the types of care considered

Could you please advise what care you would be willing to provide to your hospitalized family member?

Category of care	Type of care	Yes	No	score
**Dependency Care**	Help with eating or changing clothes			
	Help with walking or using a wheelchair			
	Help with position changes or sitting in a chair			
	Accompaniment to the sink			
**Intimate care**	Hair dressing, shaving or massage,			
	accompaniment to the toilet			
	help with using the toilet			
	help in the shower			
	emptying the urine bag or collecting urine for diuresis or other tests			
**Technical Care**	Administer medications orally,			
	Perform mouth care			
	put on or take off the nasal cannulas for oxygen or the high concentration oxygen mask			
	take the temperature			
	perform a hair blood glucose test			
Total score:				

Questionnaire for Healthcare Professionals Regarding Family Involvement In Care Of Patients Hospitalized At the Acute Assessment Unit (AAU)

Health professional code: ……………….

We invite you to share your opinion on the involvement of families in the care of hospitalized patients. This questionnaire is anonymous and will only take a few minutes to complete. Please check the box that corresponds to your answer.

Some information about you:

- **Age**: ……..........years
- **Sex**: M ☐ F ☐
- **Function**: Senior Doctor ☐ Junior Doctor ☐ Nurse ☐ Technical assistant ☐
- **Years of Experience**: ………….years
    - < 1 year ☐
    - > 1 year ☐

Questionnaire for Healthcare Professionals Regarding Family Involvement In Care Of Patients Hospitalized At the Acute Assessment Unit (AAU)

I/ Healthcare professionals' attitudes toward family involvement in care:

What do you think about family involvement in the care of hospitalized patients?

1. Involvement of families in patient care is useful. You are :

   Completely disagree ☐ Disagree ☐ Agree ☐ Completely agree ☐

2. The participation of families in patient care is essential. You are :

   Completely disagree ☐ Disagree ☐ Agree ☐ Completely agree ☐

3. The participation of the family in care provides psychological support to the patient:

   Completely disagree ☐ Disagree ☐ Agree ☐ Completely agree ☐

4. Participation in care can reduce the anguish and anxiety of the patient's family:

   Completely disagree ☐ Disagree ☐ Agree ☐ Completely agree ☐

5. Family participation in care is not dangerous for the patient:

   Completely disagree ☐ Disagree ☐ Agree ☐ Completely agree ☐

6. Family involvement in patient care does not interfere with your work:

   Completely disagree ☐ Disagree ☐ Agree ☐ Completely agree ☐

7. In general, family involvement in care does not make them hostile and aggressive towards you and your colleagues:

   Completely disagree ☐ Disagree ☐ Agree ☐ Completely agree ☐

8. You consider that family participation in patient care is a good thing for patients:

   Completely disagree ☐ Disagree ☐ Agree ☐ Completely agree ☐

9. You consider that family participation in the care of dependent patients is a good thing for families:

   Completely disagree ☐ Disagree ☐ Agree ☐ Completely agree ☐

Questionnaire for Healthcare Professionals Regarding Family Involvement In Care Of Patients Hospitalized At the Acute Assessment Unit (AAU)

II/ Healthcare professionals' opinions regarding the types of care considered

What types of care are you in favor of involving patients' families in?

Category of care	Type of care	Yes	No	score
**Dependency Care**	Help with eating or changing clothes			
	Help with walking or using a wheelchair			
	Help with position changes or sitting in a chair			
	Accompaniment to the sink			
**Intimate care**	Hair dressing, shaving or massage,			
	accompaniment to the toilet			
	help with using the toilet			
	help in the shower			
	emptying the urine bag or collecting urine for diuresis or other tests			
**Technical Care**	Administer medications orally,			
	Perform mouth care			
	put on or take off the nasal cannulas for oxygen or the high concentration oxygen mask			
	take the temperature			
	perform a hair blood glucose test			
Total score:				

Study protocol

Study Title: Opinions of Patients, Families and Healthcare Professionals on Family Involvement in the Care of Patients Hospitalized In an Acute Assessment Unit of a Moroccan University Hospital

1. Introduction - Objectives

Family involvement in the care of hospitalized patients is now well known and increasingly established in pediatric hospitals, where the presence of parents and their participation in care is an integral part of their child's hospital care as part of the Child/Parent/Caregiver triad [1].

Over the last two decades, this involvement of families in care has been increasingly studied in Western countries in the case of adults in hospital, particularly in intensive care, where the involvement of families in care has been the subject of recommendations for freer visiting [2]. Extended visiting hours are recognized as essential for involving families in patient care [3]. Additionally, open visiting policies can enhance the perceptions of families and staff, encouraging them to be more supportive of family involvement in various care activities [4].

Family involvement in inpatient care is part of the approach known as "Patient and Family Centred Care" (PFCC). The PFCC are seen as a three-legged stool, with one leg representing the carers, another representing the patient and the third representing the family. Each leg must be as important as the other in order to optimize the satisfaction of the three protagonists and the patient's progress [5].

The literature has widely addressed the opinions of patients, families, and health professionals regarding the partially passive participation of the family in care, in particular, presence at the bedside, presence during invasive procedures, and participation for medical rounds. However, few opinion surveys have been able to assess the active participation of the family in care and the types of care in which families prefer to participate [6,7]. It is recognized that the views and attitudes of healthcare professionals towards the involvement of families in care are essential for the implementation of PFCC. The adoption of a positive attitude by health professionals towards the involvement of families promotes their active participation in the care process [8]. In acute care settings, there is little evidence of family involvement in the care of adult inpatients in either Western or resource-limited countries [9]. In Morocco, a lower middle-income North African country, the participation of families in the care of their hospitalized family members is a usual practice, but is not formalized and remarkably less studied.

While several models for implementing the multiple aspects of PFCC have been proposed in Western countries, there is a notable lack of literature on the concept and practice of PFCC in the African context [10]. The aims of this study, carried out in the Acute Assessment Unit at the Ibn Sina University Hospital in Rabat, are to:

1. Evaluate the opinions of patients, families and healthcare professionals regarding family involvement in care;
2. Identify the preferences of patients, families and healthcare professionals regarding the proposed care;
3. Determine the factors associated with the preferences of patients, families and healthcare professionals regarding the proposed care.

2. Material and methods

Study design

Prospective observational study with a planned duration of eight months.

Study setting

Acute Assessment Unit (AAU) of the Ibn Sina University Hospital in Rabat.

Inclusion criteria

The study will include (1) all patients hospitalized in the AAU, (2) their families, and (3) all healthcare professionals in the AAU during the study: senior doctors, junior doctors, nurses and support staff.

Exclusion criteria

The following were excluded: (1) patients, their families and healthcare professionals who refused to take part in the study; (2) patients with missing data and (3) patients who stayed for less than 48 hours. A hospital stay of more than 48 hours was considered necessary to allow patients and their families to become familiar with the care offered and to facilitate the collection of opinions from them.

Study Variables

- **Socio-demographic variables of hospitalized patients**: (age, sex, social situation, level of education, Living conditions),
- **Clinical variables of hospitalized patients on admission:**
    - Loss of autonomy assessed by the Activities of Daily Living (ADL) score [11],
    - Level of co-morbidity assessed by the Comorbidities and Charlson Index (CCI) score [12],
    - Frailty prior to the acute episode leading to the hospitalization, assessed by the Clinical Frailty Scale (CFS) [13],
    - Probability of mortality assessed by the Simplified Acute Physiology Score II (SAPS II) calculated within the first 24 hours of admission [14],
    - Diagnosis upon admission,
    - Medical history (Previous hospitalizations, chronic disease).

- **Outcome parameters**: (length of stay in AAU, length of stay in hospital, mortality in AAU and in hospital).
- **Information about the family member(s)**:, number of family members per patient, family relationship, gender of family members, whether or not the family member are present on a

regular (daily) basis, whether or not the family members are present continuously (morning, afternoon and night).

- **Attitudes of healthcare professionals, patients and families toward family involvement in care** assessed by questionnaires based on a four-point Likert scale.
- **Opinions of healthcare professionals, patients and families** regarding the 14 types of care considered, divided into three categories: dependency-related care, intimate care and technical care.

Statistical analysis

- Quantitative variables were expressed as median with quartiles or mean ± standard deviation, and qualitative variables as percentages;
- The Student's t test and Chi 2 or Fisher's exact test will be used respectively for univariate comparison of quantitative and qualitative variables;
- Other standard statistical tests may be used depending on the results obtained.
- A univariate and multivariate analysis will be performed using an appropriate regression model to determine the factors associated with participant preferences.

3. Ethical considerations

- The study has been approved by the Biomedical Research Ethics Committee of Mohammed V University in Rabat (Reference number 41/15).
- Informed consent will be obtained from all participants, and strict measures will be implemented to ensure data confidentiality.
- The study results will be used exclusively for scientific research purposes.

Reference

1. Mastro, K.A.; Flynn, L.; Preuster, C. Patient- and family-centered care: a call to action for new knowledge and innovation. *J Nurs Adm*. **2014**; *44*, 446–51.
2. Giannini, A.; Garrouste-Orgeas, M.; Latour, J.M. What's New in ICU Visiting Policies: Can We Continue to Keep the Doors Closed? *Intensive Care Med* **2014**, *40*, 730–733, doi: 10.1007/s00134-014-3267-y.
3. Azoulay, E.; Pochard, F.; Chevret, S.; Arich, C.; Brivet, F.; Brun, F.; Charles, P.-E.; Desmettre, T.; Dubois, D.; Galliot, R.; et al. Family Participation in Care to the Critically Ill: Opinions of Families and Staff. *Intensive Care Med*. **2003**, *29*, 1498–1504. https://doi.org/10.1007/s00134-003-1904-y.
4. Garrouste-Orgeas, M.; Willems, V.; Timsit, J.-F.; Diaw, F.; Brochon, S.; Vesin, A.; Philippart, F.; Tabah, A.; Coquet, I.; Bruel, C.; et al. Opinions of Families, Staff, and Patients about Family Participation in Care in Intensive Care Units. *J. Crit. Care*. **2010**, *25*, 634–640. https://doi.org/10.1016/j.jcrc.2010.03.001.
5. Baas, L.S. Patient- and family-centered care. *Heart Lung*. **2012**, *41*, 534–535. https://doi.org/10.1016/j.hrtlng.2012.08.001
6. Liput, S.A.; Kane-Gill, S.L.; Seybert, A.L.; Smithburger, P.L. A Review of the Perceptions of Healthcare Providers and Family Members toward Family Involvement in Active Adult Patient Care Care in the ICU. *Crit Care Med*. **2016**, *44*, 1191–1197, https://doi.org/10.1097/CCM.0000000000001641.

7. Olding, M.; McMillan, S.E.; Reeves, S.; Schmitt, M.H.; Puntillo, K.; Kitto, S. Patient and Family Involvement in Adult Critical and Intensive Care Settings: A Scoping Review. *Health Expectations* **2016**, *19*, 1183–1202. https://doi.org/10.1111/hex.12402.
8. Svavarsdottir, E.K.; Sigurdardottir, A.O.; Konradsdottir, E.; Stefansdottir, A.; Sveinbjarnardottir, E.K.; Ketilsdottir, A.; Blondal, K.; Jónsdóttir, A.; Bergs, D.; Guðmundsdottir, H. The Process of Translating Family Nursing Knowledge into Clinical Practice. *J Nurs Scholarsh*. **2015**, *47*, 5–15. https://doi.org/10.1111/jnu.12108.
9. Ewart, L.; Moore, J.; Gibbs, C.; Crozier, K. Patient- and family-centred care on an acute adult cardiac ward. *Br J Nurs*. **2014**, *23*, 213–218.
10. Yakubu, K.; Malan, Z.; Colon-Gonzalez, M.C.; Mash, B. Perceptions about Family-Centred Care among Adult Patients with Chronic Diseases at a General Outpatient Clinic in Nigeria. *Afr. J. Prim. Health Care Fam. Med*. **2018**, *10*, e1–e11. https://doi.org/10.4102/phcfm.v10i1.1739
11. Katz, S.; Downs, T.D.; Cash, H.R.; Grotz, R.C. Progress in developmentof the index of ADL. *Gerontologist* .1**970**, *10*, 20–30. https://doi.org/10.1093/geront/10.1_part_1.20
12. Charlson, M.E.; Pompei, P.; Ales, K.L.; MacKenzie, C.R. A new method of classifying prognostic comorbidity in longtitudinal studies: development and validation. *J Chronic Dis*. **1987**, *40*, 373–83. https://doi.org/10.1016/0021-9681(87)90171-8
13. Rockwood, K.; Song, X.; MacKnight, C.; Bergman, H.; Hogan, D.B.; McDowell, I.; Mitnitski, A. A global clinical measure of fitness and frailty in elderly people. *CMAJ* **2005**; *173*, 489–95.
14. Gall, J.-R. A New Simplified Acute Physiology Score (Saps Ii) Based on a European/North American Multicenter Study. *JAMA* **1993**, *270*, 2957. https://doi.org/10.1001/jama.1993.03510240069035.
